# Anterior uveitis secondary to type II essential cryoglobulinemia

**DOI:** 10.1186/1869-5760-3-56

**Published:** 2013-07-25

**Authors:** Laura Nicholson, Lucia Sobrin

**Affiliations:** 1 Department of Ophthalmology, Harvard Medical School, Boston, MA, USA; 2 Massachusetts Eye and Ear Infirmary, 243 Charles Street, 12th Floor, Boston, MA 02114, USA

**Keywords:** Anterior uveitis, Cryoglobulinemia, Immunoglobulins

## Abstract

**Background:**

The purpose of this report is to describe the association of severe anterior uveitis with type II essential cryoglobulinemia.

**Findings:**

A 40-year-old male with a history of psoriatic arthritis presented with severe anterior uveitis associated with type II essential cryoglobulinemia. His uveitis, refractory to steroid treatments, was well controlled following treatments for cryoglobulinemia. The temporal association between his cryoglobulinemia and uveitis, combined with his improved visual acuity and inflammation after plasmapheresis and rituximab infusions, suggests cryoglobulinemia to be the underlying condition of his uveitis.

**Conclusions:**

To our best knowledge, this is the first reported case of anterior uveitis secondary to type II essential cryoglobulinemia.

## Findings

### Introduction

Cryoglobulinemia is a rare condition characterized by the presence of immunoglobulins that precipitate in the blood at low temperatures [1]. Type I (simple) cryoglobulinemia results from monoclonal immunoglobulins, usually IgM and IgG, and is associated with lymphoproliferative disorders. Types II and III (mixed) cryoglobulinemias involve rheumatoid factors that form complexes with the Fc portion of IgG and can be associated with chronic infectious diseases as well as autoimmune and neoplastic conditions [1]. When no underlying condition is identified, the cryoglobulinemia is termed ‘essential’. Although cryoglobulinemia is primarily known to involve the joints, kidneys, and skin, a few cases of eye manifestations have been reported [2-4]. Type I cryoglobulinemia has been associated with moderate anterior uveitis and anterior segment ischemia [2]. Isolated anterior segment ischemia without inflammation has also been demonstrated in type II cryoglobulinemia [2]. Scleritis and peripheral ulcerative keratitis have been reported with hepatitis C-related type III cryoglobulinemia [3]. One case of anterior uveitis has also been reported in a patient with cutaneous vasculitis and an unspecified type of cryoglobulinemia [4]. We describe a patient with severe anterior uveitis secondary to type II essential cryoglobulinemia. To our best knowledge, this is the first reported case of this association.

### Case report

A 40-year-old male with a history of psoriatic arthritis presented with blurred vision in both eyes. His visual acuities were 20/80 in the right eye and 20/70 in the left eye. Intraocular pressures were 20 and 18, respectively. He had 4+ anterior chamber cell according to SUN criteria [5], fine keratic precipitates, bilateral posterior synechiae, no hypopyon, and normal fundus examinations in both eyes. Fluorescein angiography demonstrated bilateral disc leakage. Concomitantly, the patient was admitted for dyspnea, leg swelling, and fever and was found to have crescentic cryoglobulinemic glomerulonephritis and pulmonary capillaritis on kidney and lung biopsies, respectively. Cryoprotein was also identified in the serum consisting of two components, an IgG kappa and an IgM kappa, consistent with type II cryoglobulinemia. The patient had negative hepatitis C serologies, was negative for syphilis and HLA-B27, and showed no evidence of tuberculosis on a chest CT scan. An extensive workup for underlying lymphoproliferative and autoimmune diseases was also negative. The patient had not been treated with any immunosuppressive therapies for his psoriatic arthritis in the past. He received hourly difluprednate drops for 3 weeks for his uveitis as well as concomitant intravenous pulse dose methylprednisolone followed by prednisone (60 mg daily) and oral cyclophosphamide (150 mg daily) for his glomerulonephritis. Despite receiving all of the above therapies for at least 2 weeks, he continued to have 4+ anterior chamber cells in both eyes. He underwent six sessions of plasmapheresis along with four intravenous rituximab infusions of 795 mg each over a 1-month period. Pulse methylprednisolone was given with the initial rituximab infusions. Over the course of these treatments, the patient’s plasma creatinine returned to normal levels, there were no plasma cryoglobulins present, and the inflammation in both eyes was resolved. His visual acuity improved to 20/20 in both eyes. At a 1-month follow-up, the patient had no anterior chamber cells, and at a 6-month follow-up, he remained taking 5 mg of prednisone with no evidence of cryoglobulinemia.

### Discussion

Although the association between psoriasis and anterior uveitis is well established [6], several aspects of this patient’s case indicate that his anterior uveitis was secondary to type II essential cryoglobulinemia rather than his psoriatic arthritis. First, there is a temporal relationship between the development of anterior uveitis and cryoglobulinemia. The patient presented with uveitis and was discovered to have type II essential cryoglobulinemia within a 2-week period. It remains possible that the cryoglobulinemia was incidental and that the timing of these conditions were unrelated. However, the patient had psoriatic arthritis for many years without developing uveitis, and his psoriatic arthritis was well controlled at the time he presented with intraocular inflammation. Second, the patient’s uveitis was refractory to a 2-week regimen of traditional treatments (topical and systemic steroids) for anterior uveitis secondary to psoriasis. In contrast, his visual acuity improved immediately following the initiation of the rituximab and plasmapheresis he received for his type II essential cryoglobulinemia. This is consistent with reports of uveitis secondary to other types of cryoglobulinemia where ocular inflammation was unresponsive to topical steroids [2,4] and oral prednisone [2], with the case of type I cryoglobulinemia-associated uveitis also being subsequently responsive to rituximab and plasmapheresis therapy [2].

There is evidence that circulating immune complexes are implicated in the pathogenesis of some uveitis cases. Animal models of serum sickness, a circulating immune complex disease, have been shown to develop cellular infiltrates in the uvea [7]. We hypothesize that the circulating immune complexes in our patient with type II essential cryoglobulinemia led to cellular infiltration in the anterior chamber in an analogous fashion to that seen in the serum sickness animal model. Thus, type II essential cryoglobulinemia should be considered in the differential diagnosis of anterior uveitis, especially in the setting of systemic manifestations of vasculitis. This is particularly important because, at least based on the few cases reported in the literature thus far, uveitis secondary to cryoglobulinemia may be refractory to traditional therapy with corticosteroids and may require more aggressive treatments like plasmapheresis and rituximab to clear the body of the culprit antibodies and restore vision.

## Competing interests

The authors declare they have no competing interests.

## Authors’ contributions

LS conceived the report and participated in writing and revising the manuscript. LN was responsible for drafting the manuscript. Both authors read and approved the final manuscript.
